# Supplementary material for: Causal interplay between lactose intolerance and gut microbiota: a combined bidirectional Mendelian randomization and in vivo validation study
Source: Front Nutr. 2026 Jun 1;13:1803337. doi: 10.3389/fnut.2026.1803337 (PMC13265576; doi:10.3389/fnut.2026.1803337)
Supplement: Supplementary file 1 [file Data_Sheet_1.zip › supplementary materials/Forward/forest plot/ebi-a-GCST90027794.finngen_R12_E4_LACTONAS.pdf]

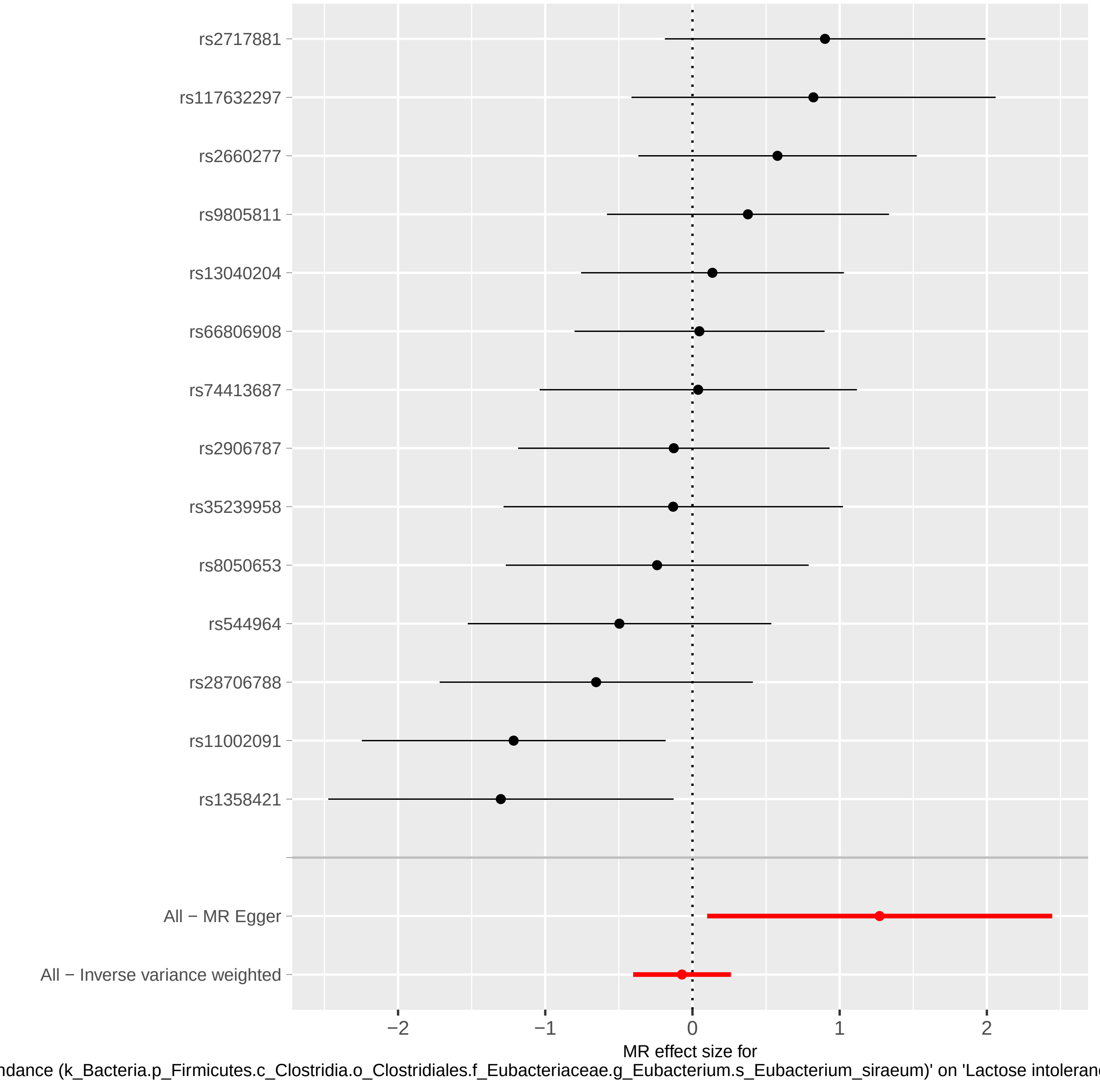
rs2717881
rs117632297
rs2660277
rs9805811
rs13040204
rs66806908
rs74413687
rs2906787
rs35239958
rs8050653
rs544964
rs28706788
rs11002091
rs1358421
All − MR Egger
All − Inverse variance weighted
−2
−1
0
1
2
MR effect size for
ndance (k_Bacteria.p_Firmicutes.c_Clostridia.o_Clostridiales.f_Eubacteriaceae.g_Eubacterium.s_Eubacterium_siraeum)' on 'Lactose intoleran
